# Forty years of research in animal models of human diseases and outstanding achievements in supporting human life and health

**DOI:** 10.1002/ame2.12146

**Published:** 2021-01-23

**Authors:** Chuan Qin

**Affiliations:** ^1^ Chinese Association for Laboratory Animal Sciences Beijing China; ^2^ Institute of Laboratory Animal Science Chinese Academy of Medical Science and Comparative Medicine Center Peking Union Medical College Beijing China

On November 19‐20, 2020, the 2nd Longtan Science Conference and the 40th anniversary celebrations of the Institute of Laboratory Animal Sciences, Chinese Academy of Medical Sciences & Comparative Medicine Center, Peking Union Medical College (hereinafter referred to as "ILAS") were held in Beijing. The conference was jointly organized by the Institute and the Chinese Association for Laboratory Animal Sciences.

Professor Chuan Qin, President of Institute of Laboratory Animal Science, Chinese Academy of Medical Science and Peking Union Medical College, President of Chinese Association for Laboratory Animal Sciences.

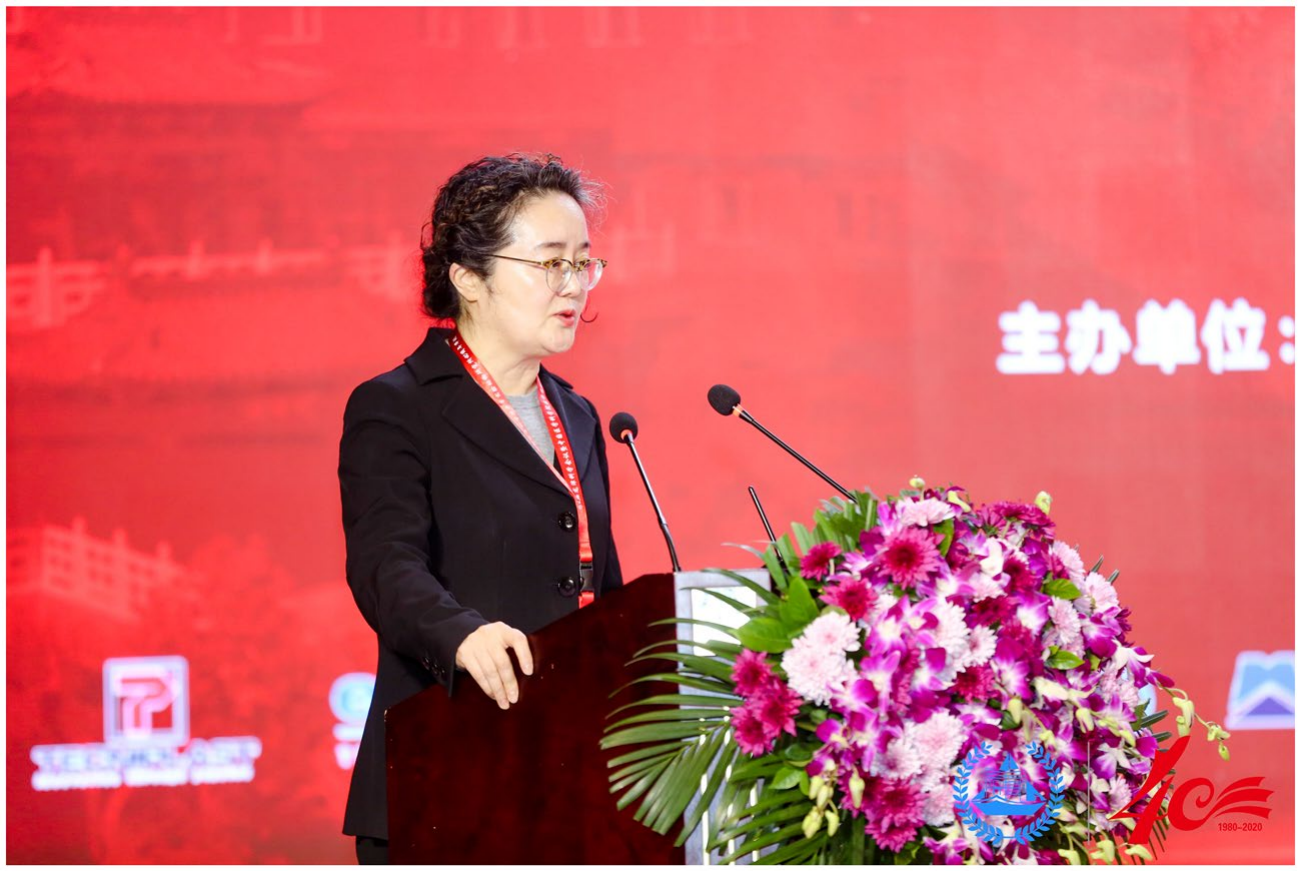

Laboratory animal science is a key support for and an important component of life science and health research. Since its establishment in 1980, the mission of ILAS has been to foster the development of disciplines and resources, basic research, and technical training.

Following the founding of the first animal rooms 40 years ago, generations of ILAS graduates have made dedicated efforts to develop laboratory animal science research and establish the discipline of comparative medicine. Through their unremitting efforts, ILAS has set up the largest human disease animal model resource center in China, established animal models of infectious diseases in all previously targeted areas in the fight against infectious diseases, and built a national system of laboratory animal standards.

Since 2003, ILAS has entered a period of rapid development. With the emergence of SARS and continuing with the emergence of COVID‐19, ILAS has been tasked with the important mission of establishing disease animal models, and has made great contributions to the prevention and control of infectious diseases worldwide.

The development of ILAS over the past 40 years would not have been possible without the support of other agencies. At the opening ceremony, guests from CPPCC, the National Health Commission, the Ministry of Science and Technology, and the Chinese Academy of Medical Sciences & Peking Union Medical College offered congratulations and high praise for the efforts made by ILAS to accelerate innovation in medical sciences, promote original drug and vaccine research, and ensure public health and safety.

Over 40 years, ILAS has fostered the development of dedicated and innovative scientists. The conference issued a number of awards celebrating the achievements of these leading scientists: "Epidemic research pioneer award", "40th anniversary research achievement award", "Lifetime achievement award", "Outstanding contribution award" and "Outstanding young scholar award".

Lifetime achievement award: Professor Lu Yaozeng

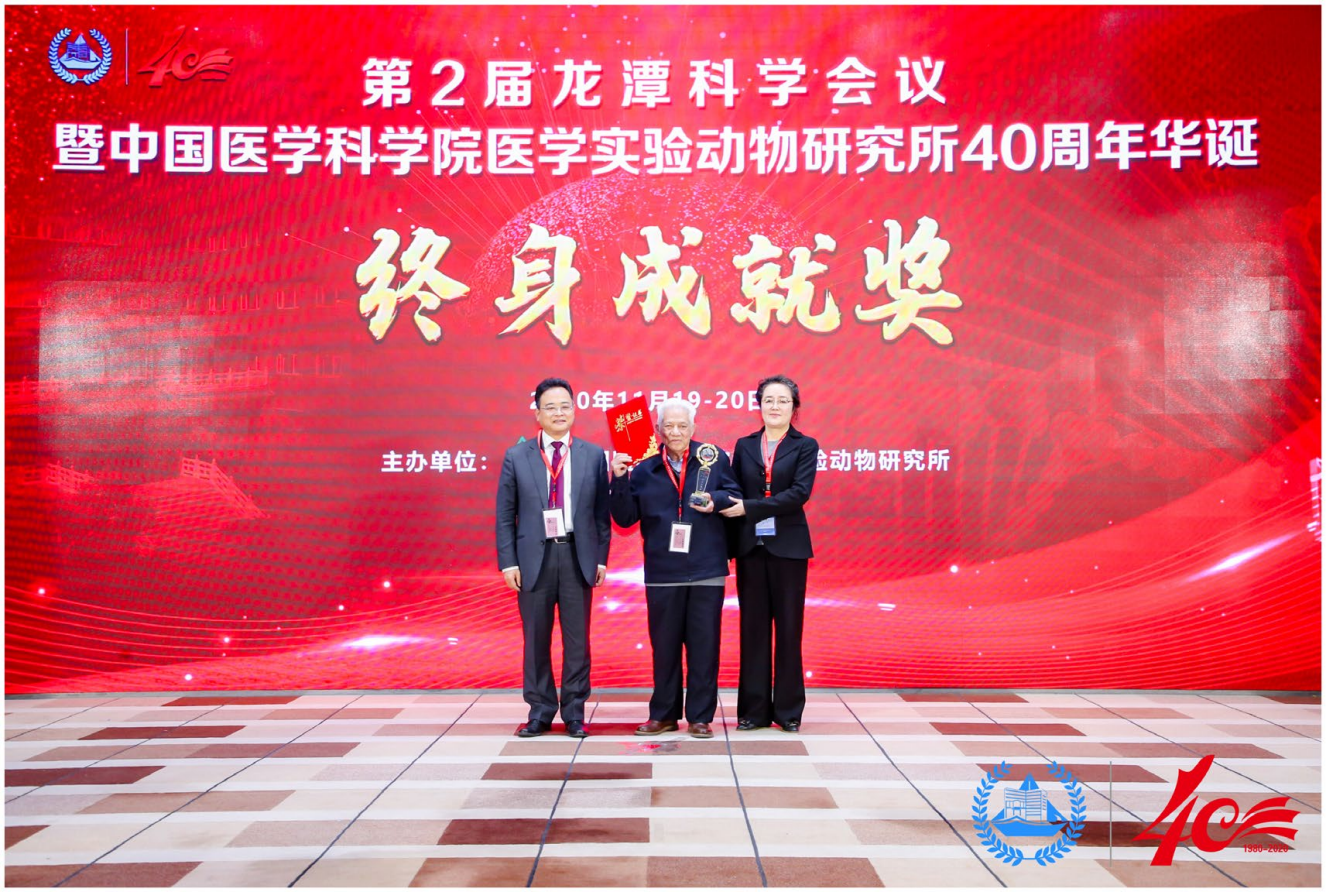

The celebrations of the 40th anniversary of ILAS were held jointly with the annual Longtan Science Conference. At the conference, 15 academicians and experts gave academic lectures on medical education, scientific and technological innovation, animal model research, etc., with a focus on promoting the development of research in laboratory animal science, comparing medicine and life science, and better safeguarding of human health through cutting‐edge academic exchanges. As part of its 40th anniversary events, ILAS has also organised a series of online academic lectures at home and abroad between November, 2020 and January, 2021, to enhance academic exchanges and expand academic horizons, with speakers including Professor Fuad Iraqi (Tel Aviv University, Israel), Professor Grant Morahan (University of Western Australia), and Professor Erwan Bezard (University of Bordeaux).

Lifetime achievement award: Professor Lu Yaozeng

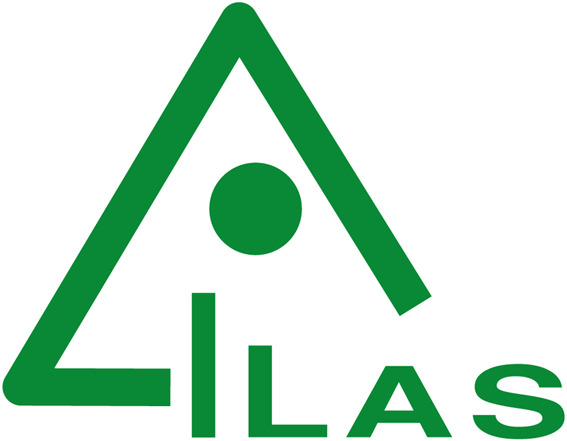

Institute of Laboratory Animal Sciences,

Chinese Academy of Medical Sciences

http://www.cnilas.org/

renshichu@cnilas.org

86‐(0)10‐67776050

86‐(0)10‐67776529

the Website of ILAS 40th anniversary

http://www.cnilas.org/suoqing40/

The Website of *Nature* for ILAS 40th anniversary

Nature website https://www.nature.com/articles/d42473‐020‐00447‐8

